# The Economic Burden of Violence against Children in South Africa

**DOI:** 10.3390/ijerph14111431

**Published:** 2017-11-22

**Authors:** Xiangming Fang, Xiaodong Zheng, Deborah A. Fry, Gary Ganz, Tabitha Casey, Celia Hsiao, Catherine L. Ward

**Affiliations:** 1 College of Economics and Management, China Agricultural University, No. 17 Qinghuadong Road, Haidian District, Beijing 100083, China; zhengxd@cau.edu.cn; 2 School of Public Health, Georgia State University, 140 Decatur Street, Atlanta, GA 30303, USA; 3 Moray House School of Education, St John’s Land, 2.02, Holyrood Road, Edinburgh EH8 8AQ, Scotland; Debi.Fry@ed.ac.uk (D.A.F.); Tabitha.Casey@ed.ac.uk (T.C.); 4 Department of Psychology and Safety and Violence Initiative, University of Cape Town, Rondebosch 7701, South Africa; garyganz@gmail.com (G.G.); Catherine.Ward@uct.ac.za (C.L.W.); 5 Save the Children South Africa, 2nd Floor SAQA House, 1067 Arcadia Street, Hatfield 0028, South Africa; CHsiao@savethechildren.org.za

**Keywords:** economic burden, violence against child, South Africa, disability-adjusted life-year (DALY)

## Abstract

The purpose of this study was to estimate the economic burden of violence against children in South Africa. We assembled summative estimates of lifetime prevalence, calculated the magnitude of associations with negative outcomes, and thereby estimated the economic burden of violence against children. According to our calculations, 2.3 million and 84,287 disability-adjusted life-years (DALYs) lost in South Africa in 2015 were attributable to nonfatal and fatal violence against children, respectively. The estimated economic value of DALYs lost to violence against children (including both fatal and nonfatal) in South Africa in 2015 totalled ZAR173 billion (US $13.5 billion)—or 4.3% of South Africa’s gross domestic product (GDP) in 2015. In addition, the reduced earnings attributable to childhood physical violence and emotional violence in South Africa in 2015 were ZAR25.2 billion (US $2.0 billion) and ZAR9.6 billion (US $750 million), respectively. In addition, South Africa spent ZAR1.6 billion (US $124 million) on child care and protection in fiscal year 2015/2016, many of which costs are directly related to violence against children. This study confirms the importance of prioritising violence against children as a key social and economic concern for South Africa’s future.

## 1. Introduction

Violence against children exists in every country in the world, cutting across culture, class, education, income and ethnic origin. Sadly, South Africa is no exception. Childhood violence can have lifelong adverse health, social and economic consequences for survivors and the society. Given the high prevalence of violence against children and the many negative short- and long-term consequences, the economic costs of violence against children may be substantial. Given such costs, violence against children is not only a human rights and moral issue, but also an economic one.

Previous burden of violence against children studies have found significant impacts on children’s and adults’ mental and physical health, employment and education, as well as increasing risk factors for experiencing other forms of violence [1]. A burden study in the East Asia and Pacific region found that violence against children costs the region 2% of their GDP [2]. Estimates of the economic burden have also been published for a few countries individually such as the United States [3], Australia [4], and China [5], but are lacking in most countries and regions of the world, including Africa. In the US, for instance, it was estimated that the average lifetime cost per victim of nonfatal violence against children was US $210,012 in 2010; whereas the estimated average lifetime cost per death was US $1,272,900. Together, the total lifetime economic burden resulting from new cases of fatal and nonfatal violence against children in the US in 2008 alone was estimated to be approximately US $124 billion [3].

No similar data exists for the economic burden of violence against children in Africa, making this the first study to explore this burden on the continent. While studies of the prevalence and incidence of violence against children provide authorities with information relevant to service planning—for instance, how many staff members would be needed to deal with new reports of child maltreatment in a year—this is often not a sufficient rationale for policymakers to develop services in contexts (like low- and middle-income countries, such as South Africa) where there are competing urgent priorities for the scarce resources within national budgets. Information on how much violence against children costs the economy can provide that rationale, if costs of the violence outweigh the costs of prevention and intervention services.

The purpose of this study, therefore, was to estimate the economic burden of violence against children in South Africa. Through assembling summative estimates of lifetime prevalence and calculating the strength of associations with negative outcomes, we estimated the economic burden of violence against children. The data generated in this study is intended to advance the awareness of policymakers of the economic impact of violence against children and therefore support budget allocations and investments in this regard.

## 2. Materials and Methods

Three steps were used in the estimation of the economic burden of violence against children in South Africa.

### 2.1. Step One—Systematic Review of Prevalence and Consequences

A systematic review was conducted to identify studies reporting on the prevalence and consequences of violence against children in South Africa using the Africa-Wide Information, MEDLINE, PsycINFO, CINAHL, ERIC, SocINDEX and Embase databases. Peer-reviewed and non-peer-reviewed journal articles, research reports and other ‘grey’ literature reporting prevalence and/or consequences of violence with a geographic focus in South Africa, that were published between January 2000 and December 2015, were included. Key child protection researchers and organisations in South Africa were also contacted to identify additional studies. The reference lists of key narrative reviews on violence against children in South Africa and the region were also scanned for additional studies, and a manual search of eight international and national journals was also conducted. The eight journals include *Child Abuse and Neglect*, *Child Maltreatment*, *Child Abuse Review*, *Journal of Interpersonal Violence*, *South African Medical Journal*, *South African Journal of Psychology*, *African Journal of Psychiatry*, and *Journal of Child and Adolescent Mental Health*.

Studies were included if they reported the prevalence/incidence of sexual, physical or emotional violence against children, neglect, witnessing family violence, community violence, bullying, gang violence and other forms of violence affecting children in South Africa. For prevalence/incidence studies additional inclusion criteria were: (i) Participants were recruited from a student or general population; (ii) quantitative methods were used to estimate the prevalence/incidence of the violence during childhood (e.g., younger than 18 years); (iii) the study reported the prevalence or incidence of violence against children; and (iv) the recorded violence had been reported directly by the victims or by parents. For the review of outcomes studies, additional inclusion criteria were: (i) primary research that explored the relationship between at least one form of violence against children and its impact on employment, education, mental health, physical health, health behaviours, aggression, violence, criminality, exposure to further violence, formal and informal care and service use; (ii) included the calculation of odds ratios (ORs) or relative risks (RRs) or marginal effects (MEs) disaggregated by the type of violence; and (iii) did not sample on the basis of the presence of any specified outcome—since this would invalidate the calculation of an OR or RR or MR for that outcome. The review utilised both free text and controlled vocabulary of subject heading and keyword searches to identify articles and grey literature via the electronic databases. To provide the broadest coverage of articles, the initial search term consisted of: (1) population (e.g., children); (2) type of maltreatment (e.g., physical abuse); and (3) South Africa. Five search strings were utilised in total. To ensure accuracy, the searches were conducted by two researchers and results compared; papers were retained if there was consensus between the two reviewers about inclusion, and any remaining questions were resolved by consultation with the rest of the team if necessary. Relevant data from included articles was then extracted into an Excel spreadsheet. The full details of the search strategy, search terms used, inclusion criteria, excluded articles, a full description of all violence against children studies, and data extraction can be found elsewhere [6].

Given that there were too few studies to yield reliable estimates on the consequences of witnessing parental violence and exploitation, these forms of violence against children were not included in this study. This study only focuses on four major types of violence against children: physical violence, sexual violence, emotional violence, and neglect. We also did not distinguish violence perpetrated by children from that perpetrated by adults: our definition of violence against children was based solely on whether the victim was a child, in accord with the Optimus Study South Africa [7]. The systematic review identified a total of 65 studies. For consequences, a total of 24 studies met the inclusion criteria: 10 measured the relationship between violence against children and interpersonal violence, 4 measured anxiety, 3 measured self-harm, 3 measured alcohol abuse, 3 measured depression, 3 measured sexually transmitted diseases, 2 measured drug abuse, 1 measured HIV, and 5 measured other types of outcomes such as unwanted pregnancy or high school drop-out [6]. No studies reported odds ratios (ORs) or relative risks (RRs) for the effects of violence against children on employment, formal and informal care, or service use (e.g., health care services, criminal justice services). Given that the existing literature mostly investigated the impacts of violence against children on health outcomes and health risk behaviours, there is no sufficient information from the systematic review to estimate the economic costs associated with the consequences of violence against children beyond health outcomes and health risk behaviours. Thus, following the methods used by previous studies [2,5], we estimated the economic burden of violence against children by first estimating the disability-adjusted life years (DALYs) lost from health outcomes and health risk behaviours attributable to childhood violence and then converting the DALY losses into a monetary value using a human capital approach, assuming that one DALY is equal to the country’s per-capita GDP.

Following a systematic review of the prevalence literature, we began a meta-analysis to determine the prevalence rates. However, at the same time, the results from the Optimus Study South Africa [7] were released—these provide the first nationally representative figures of different forms of violence against children in South Africa. Since nationally representative studies provide more accurate data than those that can be pieced together through a meta-analysis, the Optimus Study data [7] were used in subsequent analyses.

### 2.2. Step Two—Calculation of Population Attributable Fractions (PAFs)

Population Attributable Fractions (PAFs) were used to estimate the proportion of morbidity or mortality attributable to a risk factor. All PAF formulas require: (1) Relative risk (RR) of a disease or outcome (e.g., depression) given exposure to a risk factor (violence against children), or an odds ratio (OR) which can be converted into an approximate estimate of the relative risk (RR); and (2) a measure of prevalence. To calculate a population attributable fraction, it is necessary to know the prevalence of a risk factor—e.g., violence against children—and the relative rate for the disease or outcome of interest—e.g., depression—given exposure to that risk factor. In order to match the outcomes with the available Global Burden of Disease categories, the outcomes were further limited to: alcohol abuse, drug abuse, sexually transmitted diseases (STDs), HIV, interpersonal violence, self-harm and mental disorder—including depression and anxiety. For each of these outcomes, we attempted to calculate a population attributable fraction for each form of violence against children for which we had data.

Studies used to calculate ORs and RRs for violence against children in terms of a number of outcome relationships are presented in Table A1. Table A2 in the Appendix A presents the ORs for health outcomes associated with childhood violence that were found from the systematic review. In addition to the findings from the systematic review regarding the outcomes of violence, we conducted additional data analyses to explore outcomes of violence against children based on the Cape Area Panel Study (CAPS). The CAPS follows the lives of a large and representative sample of adolescents in Cape Town as they undergo the multiple transitions from adolescence to adulthood. The CAPS started in 2002 and ended in 2009, and includes five successive waves of survey. The study investigated the multidimensional nature of the lives of the young men and women—educational, psychological, familial, sociological, economic, and community.

Based on the data from the CAPS, generalised linear models (for binary outcome variables) or linear regression (for continuous outcome variables) were used to estimate the associations between the different types of violence against children (childhood emotional violence and physical violence) and the related young adult consequences and risk behaviours: violence perpetration, wages earned, alcohol use, drug use, obesity, and mental health. The Heckman two-stage method was used to correct for the selection bias (i.e., people who work are selected non-randomly from the population) and to estimate the marginal effect of violence against children on wages. All these regressions were controlled for socio-demographic or biological confounding factors: sex, race, respondent’s education, marital status, childhood family poverty, childhood family size, childhood family structure, and mother’s education. The Stata software (prefix SVY) was used to control for the survey design effects of individuals clustered in sampling units of enumeration areas (EAs) and stratification of major population groups in Cape Town—black African, coloured, and white.

If only the unadjusted ORs for a study were available, we produced corresponding estimates of adjusted ORs using the ratios between adjusted and unadjusted ORs reported for other studies [8]. As most studies included in the systematic review reported ORs but not RRs, RRs had to be estimated from the ORs using a simple formula [9]. The secondary data analyses based on the CAPS generated RRs directly, using generalised linear models (See Table A3 in the Appendix A).

Finally, for each type of violence, the estimated RRs were grouped according to outcomes and then combined using meta-analysis with sample size weighting.

### 2.3. Step Three—Estimating the Costs to South Africa of Violence against Its Children

In the final step, we estimated the costs to the economy of violence against South African Children using a prevalence-based approach. Cost categories available from the existing data were estimated as follows:

- Disability-Adjusted life years (DALYs) lost were calculated for both fatal violence, and for the physical and mental health outcomes, and health risk behaviours, that could be attributed to nonfatal violence against children;
- Reductions in earnings attributable to physical and emotional violence against children;
- Costs to the government child protection system.

#### 2.3.1. Monetary Value of DALY Loss

Based on the findings from the literature review and data analyses, we first estimated monetary value of DALY loss attributable to nonfatal violence against children. Following the work of the World Health Organization (WHO) [10] and Brown [11], we estimated the DALYs lost—due to violence against children-attributable physical and mental health outcomes and health-risk behaviours—and then estimated the monetary value of those DALYs in 2015 South African Rand (ZAR).

For each of the main types of violence against children that we considered, a population attributable fraction for an outcome of interest was multiplied by the estimate of the number of the DALYs expected to be lost because of that outcome. Population attributable fractions of our selected health and behavioural outcomes (alcohol abuse, drug abuse, sexually transmitted diseases (STDs), HIV, interpersonal violence, self-harm, serious mental illness, depression and anxiety) were matched to definitions of “alcohol use”, “drug use”, “STDs excluding HIV“, “HIV/AIDS”, “interpersonal violence”, “self-harm”, “mental disorders”, “depressive disorders”, and “anxiety disorders” from the Global Burden of Disease Study 2015 (GBD 2015) (Available at http://ghdx.healthdata.org/gbd-results-tool [12]).

Given the possible co-morbidity between childhood violence and other health outcomes, DALY data was only used for those aged 15+ to estimate disease-induced DALY losses. This was to avoid the possibility of diseases preceding the occurrence of childhood violence. To avoid double counting, the contribution of the cause categories (e.g., self-harm, HIV/AIDS) to DALY loss under a given risk factor (e.g., drug use) was removed, if PAFs for these cause categories (e.g., self-harm, HIV/AIDS) were available separately.

Second, the DALYs lost from fatal cases of violence against children were calculated as the number of child deaths multiplied by a loss function specifying the years lost for deaths as a function of the age at which death occurs [13]. Since the loss function is intended to represent the maximum life span of an individual in good health, who is not exposed to avoidable health risks, or severe injuries, and receives appropriate health services, the 2015 Global Burden of Disease study chose to base this on the frontier national life expectancy projected for the year 2050 by the World Population Prospects 2012 [14]; we follow this approach here.

To convert the DALY losses into a monetary value, a method employed by WHO [10] and Brown [11] was used. This method assumes that one DALY is equal to the country’s per-capita GDP. In other words, it is assumed that a year lost due to either disability or mortality (one year lived with disability or one year of life lost) is a year lost from the productive capacity of a country’s economy and can therefore, on average, be approximated by the per capita GDP; the “human capital” approach to valuing DALYs. Data on 2015 population, GDP, and GDP per capita for South Africa were obtained from Statistics South Africa (Available at http://cs2016.statssa.gov.za/ [15]).

#### 2.3.2. Reductions in Earnings

The Cape Area Panel Study is a panel study of young people in Cape Town, South Africa. It includes data on physical and emotional violence against the participants, as well as their earnings in adulthood. Using this, we were able to estimate the percentage reduction in adulthood earnings that might be attributable to these two forms of violence against children. Using data from the Optimus Study South Africa [7], we estimated how many people in the labour force had sufferance of physical or emotional violence in their lifetimes. These two pieces of data were then combined to estimate the total productivity loss in South Africa in 2015 attributable to physical and emotional violence against children.

#### 2.3.3. Child Welfare Costs

Services provided to child victims of violence also have a cost associated with them. In South Africa, costs of welfare services are reported in documents available from the National Treasury; we used these data to estimate the costs of providing these necessary services (see Table 1).

This cost under-estimates the direct costs, for several reasons: (1) It only includes what is currently spent on services, not what would be spent if all children who were victims of violence actually received services; and (2) It only includes welfare services, and no other services that child victims may need (for instance, costs of policing violence against children, taking cases through the courts, or physical or mental health services).

## 3. Results

### 3.1. Monetary Value of DALYs Lost from Nonfatal Violence against Children

Prevalence of each type of nonfatal violence against children (see Table 2), and the relative risks and population-attributable fractions (Table 3), were used together to calculate the DALYs lost to each type of violence, and hence the economic cost associated with the DALYs (Table 4).

Although only a limited number of health outcomes were considered, an estimated 375,097, 1,172,331, 636,434, and 93,804 of DALYs lost in South Africa in 2015 were attributable to childhood sexual violence, physical violence, emotional violence, and neglect, respectively. The estimated economic value of these lost DALYs was 27.4, 85.7, 46.5, and 6.9 billion South Africa Rand (0.7%, 2.1%, 1.2%, and 0.17% of GDP), respectively.

Adding up the economic value of DALY loss across different types of violence against children, 2.3 million DALYs lost in South Africa in 2015 were attributable to violence against children, a full 32% of those attributed to HIV/AIDS in 2015. The estimated economic value of DALYs lost to violence against children in South Africa in 2015 totalled ZAR166 billion (4.2% of the 2015 GDP in South Africa).

### 3.2. Economic Cost of Fatal Violence against Children

An estimated 1018 child homicides occurred in 2009 in South Africa, broken down by age group as follows: 405 cases in the 0–4 years age group, 87 in the 5–9 years age group, 110 in 10–14 years age group, and 416 in 15–17 years age group [17]. This was the only study of child homicides that we could identify, and we therefore assumed that the number of child homicides in 2015 equalled to that in 2009. In order to estimate DALYs, we needed the mean ages of the children who had died; to estimate this, we used the median age of the age group as the mean age of victims. Thus we estimated the mean age of the 405 child victims in the 0–4 age group to be 2, the mean age in the 5–9 age group to be 7, 12 for the 10–14 age group, and 16 for the 15–17 age group.

The WHO standard life table for years of life lost [13] provides standards for the expected years of life lost for a death at a particular age. We used this to estimate years of life lost at ages 2, 7, 12 and 16 to be 90.01, 85.02, 80.03, and 76.04, respectively. DALYs lost from the 1018 child deaths was then estimated to be 84,287, by multiplying the number of deaths in each age group by the corresponding years of life lost for the deaths at the mean age of the age group (90.01 × 405 + 85.02 × 87 + 80.03 × 110 + 76.04 × 416). The estimated economic value of these lost DALYs was 6.2 billion South Africa Rand (0.16% of South Africa’s GDP).

### 3.3. Reduced Earnings

Our analyses of the Cape Area Panel Study data allowed us to estimate that physical and emotional violence against children on average reduce victim monthly earnings by 11.7% and 9.2%, respectively. According to Statistics South Africa [18], the median monthly earnings for South Africa for 2014 (including employees, employers and own-account workers) was ZAR3120. We then used the Consumer Price Index to adjust this figure 2015 Rand values, yielding an estimated median monthly earning figure of ZAR3262. Combining these two pieces of data, physical and emotional violence against children on average therefore reduce victim monthly earnings by ZAR382 (3262 × 11.7%) and ZAR300 (3262 × 9.2%), respectively.

According to the weighted data from the self-administered questionnaire in household surveys that formed part of the Optimus Study South Africa [7], 26.1% of the respondents reported lifetime experience of physical violence, and 12.6% reported some experience of emotional violence in their lifetimes. In 2015, the average size of the labour force was 21,084,500 in South Africa (Available at http://cs2016.statssa.gov.za/ [15]). We then calculated how many people in the labour force in 2015 were lifetime victims of childhood physical violence (5,503,055; 21,084,500 × 26.1%) and lifetime victims of childhood emotional violence (2,656,647; 21,084,500 × 12.6%).

The total monthly productivity loss attributable to physical and to emotional violence against children in South Africa in 2015 could thus be estimated at ZAR2,100,262,762 (5,503,055 × 382) and ZAR797,270,391 (2,656,647 × 300), respectively. The corresponding annual figures could be achieved by multiplying these estimates by 12, so that the total productivity loss in South Africa for the year of 2015 attributable to physical and emotional violence against children were ZAR25.2 (0.63% of GDP) and ZAR9.6 billion (0.24% of GDP), respectively.

### 3.4. Child Welfare Costs

Together, the nine South African provinces in South Africa spent ZAR1.58 billion (0.04% of GDP) on child care and protection in fiscal year 2015/2016.

## 4. Discussion

This is the first study to estimate the economic cost of violence against children in South Africa. Data from previous studies, and in particular the Optimus Study South Africa [7], shows that violence is a common experience for South African children; our work shows that this violence is not only a human rights issue, but an economic one: violence against children costs South African society in terms of both DALYS and money. According to our calculations, 2.3 million and 84,287 DALYs lost in South Africa in 2015 were attributable to nonfatal and to fatal violence against children, respectively. Our estimates of DALYs lost to nonfatal violence against children are greater than the corresponding South African estimates for diabetes mellitus (1.1 million DALYs lost) and cardiovascular diseases (2.1 million DALYs lost) (Available at http://ghdx.healthdata.org/gbd-results-tool). HIV/AIDS was the leading cause of DALYs lost in 2015 in South Africa (Available at http://ghdx.healthdata.org/gbd-results-tool): our figures for DALYs lost to non-fatal violence against children led to the loss of 32% of the DALYs lost to HIV/AIDS. The estimated economic value of DALYs lost to violence against children (including both fatal and nonfatal) in South Africa in 2015 totalled ZAR173 billion (US $13.5 billion)—or 4.3% of South Africa’s GDP in 2015. These were not the only costs. The reduced earnings attributable to physical and emotional violence against children in South Africa in 2015 were ZAR25.2 billion (US $2.0 billion) and ZAR9.6 billion (US $750 million), respectively. Despite these high costs to the economy, South Africa spent only ZAR1.6 billion (US $124 million) on child care and protection in fiscal year 2015/2016.

There are of course limitations in our work. In particular, these estimates suffer from several major gaps in the evidence base and data available in South Africa. PAFs may be sensitive to small changes in prevalence and RR, and most of the studies used for calculating PAF did not have representative samples, used different samples and approaches to sampling, different definitions of violence against children, and different measures. The implications of these differences can be significant when multiplied by an aggregate outcome. Although we carefully reviewed all input data to select appropriate studies, our results are inevitably based on what data is available. This problem is not limited to research into violence against children; such challenges generally emerge in any research that draws on a variety of secondary data sources.

Although the DALYs measure has made a central contribution to the comparative assessment of disease burden, it is important to note that the measure has been the subject of some debate [19,20,21]. For instance, the validity and universality of the disability weights [20,21] have been questioned, and standard life expectancy figures may overestimate DALYs saved when actual or local life expectancy is shorter [22]. Furthermore, DALYs are a global generic disability index, but the relative weights for each health state/condition may be substantially different in any single country and lead to quite different results. Thus, the DALY results should be interpreted with caution.

Ideally, studies of this nature would account for poly-victimisation, or repeat victimisation—a common phenomenon in violence against children, and one which may provide the greater part of the association between victimisation and consequences [23]. Unfortunately, based on the studies available to us, we were not able to take this into account, which may have led to over-estimation of the aggregate economic burden of violence against children.

Other factors known to confound the association between exposure to violence and later outcomes (for instance, genetic and other family factors) [2,24] were also not taken into account in the studies available to us, which again could have resulted in an over-estimation of the PAFs. In addition, most of the studies of consequences that we used measured both violence exposure and the consequences by self-report in cross sectional studies, and retrospectively, and this too may lead to either over- or under-estimation. Further, because the studies used self-report, they only reported ORs. Approximate RRs needed for PAF were calculated with from ORs with a simple formula [9], but because the majority of studies were based on small sample sizes, the RRs generated from these studies may not be generalisable to the entire population and the resultant PAFs could be biased up or down. We used meta-analysis with sample size weighting to alleviate these problems, but it cannot fully eliminate this bias.

Considering all these limitations together, we suggest that the burden estimates derived from our work are likely to under-estimate the true situation. The studies of health outcomes available to us for estimating the burden of violence against children only addressed a small number of health outcomes, and our work therefore does not include other serious effects of violence against children (for instance, its association with obesity, itself associated with increased morbidity and mortality) [25]. Costs that we were not able to include in our models included the costs of poor educational outcomes; increased formal and informal care; higher levels of healthcare utilisation; criminal behaviour; reproductive health problems; and chronic diseases such as diabetes, heart disease and cancer.

## 5. Conclusions

This study reveals that the economic burden of violence against children in South Africa is substantial, and thus confirms the importance of prioritising violence against children as a key social and economic concern for South Africa’s future development. Policy-makers are urged to consider the huge economic cost, as well as the human cost, of the lifetime impacts of violence against children in their budget allocations. It provides a strong indication that resources directed towards preventing violence against children are a vital investment that could save lives, could prevent much agony for children, and increase the economic dividend that lies latent in the country’s children.

## Figures and Tables

**Table 1:** Cost of child care and protection.

Province	Revised Estimate of Child Care and Protection (2015/2016)—ZAR Thousand
Eastern Cape	216,512
Free State	78,284
Western Cape	175,376
North West	55,023
Gauteng	507,563
KZN	324,436
Northern Cape	36,687
Mpumalanga	54,092
Limpopo	133,190
National total	1,581,163

Source: National Treasury: http://www.treasury.gov.za/documents/provincial%20budget/2016/7.%20EPRE%20standardised%20tables%20in%20Excel%20format/Default.aspx [16].

**Table 2:** Prevalence of violence against children.

Type of Violence against Children	Prevalence Rate (%)
Contact sexual violence	7.2%
Males	6.1%
Females	8.5%
Physical violence	26.1%
Males	24.0%
Females	28.7%
Emotional violence	12.6%
Males	9.7%
Females	16.2%
Neglect	12.2%
Males	9.8%
Females	15.1%

Source: Artz, L. et al., (2016). Optimus Study South Africa: Technical Report [7].

**Table 3:** Population attributable fractions (PAFs) and relative risks (RRs) for health outcomes associated with violence against children.

Type of VAC	SMI		Depression		Anxiety		Alcohol Abuse		Drug Abuse		STDs		HIV		Interpersonal Violence		Self-Harm	
	RR	PAF	RR	PAF	RR	PAF	RR	PAF	RR	PAF	RR	PAF	RR	PAF	RR	PAF	RR	PAF
Sexual violence																		
Total	-	-	-	-	1.83	0.06	-	-	3.23	0.14	1.4	0.03	-	-	-	-	2.84	0.12
Males	-	-	-	-	-	-	2.2	0.07	-	-	-	-	-	-	1.5	0.0296	-	-
Females	-	-	1.73	0.06	-	-	2.25	0.1	-	-	2	0.08	1.6	0.05	1.94	0.074	-	-
Physical violence																		
Total	1.41	0.1	-	-	1.57	0.13	1.55	0.13	1.46	0.11	-	-	-	-	1.18	0.0449	2.13	0.23
Males	-	-	-	-	-	-	-	-	-	-	-	-	-	-	1.35	0.0775	-	-
Females	-	-	-	-	-	-	-	-	-	-	-	-	1.97	0.22	1.48	0.1211	-	-
Emotional violence																		
Total	1.38	0.05	-	-	1.86	0.1	1.35	0.04	1.41	0.05	-	-	-	-	1.27	0.0329	2.35	0.15
Males	-	-	-	-	-	-	1.33	0.03	-	-	-	-	-	-	-	-	-	-
Females	-	-	-	-	-	-	-	-	-	-	-	-	1.86	0.12	-	-	-	-
Neglect																		
Total	-	-	-	-	1.73	0.08	-	-	-	-	-	-	-	-	-	-	-	-
Males	-	-	2.9	0.16	-	-	-	-	1.45	0.04	-	-	-	-	-	-	-	-
Females	-	-	1.66	0.09	-	-	2.12	0.14	-	-	1.39	0.06	-	-	-	-	-	-
Witnessing family violence																		
Total	-	-	-	-	1.59	0.13	-	-	-	-	-	-	-	-	-	-	-	-
Males	-	-	-	-	-	-	-	-	-	-	-	-	-	-	1.86	0.1591	-	-
Females	-	-	-	-	-	-	-	-	-	-	-	-	-	-	1.71	0.1648	-	-

Notes: SMI, serious mental illness; STD, sexually transmitted disease; - indicates not applicable.

**Table 4:** Estimated disability-adjusted life-years (DALYs) and economic value lost to childhood violence in 2015.

Health Outcomes	Sexual Violence		Physical Violence		Emotional Violence		Neglect		Aggregate Costs	
	DALY Loss	Economic Value (Million Rand)	DALY Loss	Economic Value (Million Rand)	DALY Loss	Economic Value (Million Rand)	DALY Loss	Economic Value (Million Rand)	DALY Loss	Economic Value (Million Rand)
Serious mental illness			90,597	6619	42,824	3129			133,420	9748
Depression	14,127	1032					48,008	3507	62,135	4540
Anxiety	7941	580	18,236	1332	13,767	1006	11,515	841	51,459	3760
Alcohol abuse	70,520	5152	119,261	8713	40,128	2932	29,717	2171	25,9626	18,969
Drug abuse	18,122	1324	14,041	1026	6434	470	3604	263	42,201	3083
STDs	1106	81					960	70	2066	151
HIV	181,841	13,285	816,045	59,621	45,8238	33,479			1,456,124	106,386
Interpersonal violence	36,764	2686	45403	3317	33,290	2432			115,457	8435
Self-harm	44,677	3264	86984	6355	55,520	4056			187,180	13,676
Total	375,097	27,405	1,172,331	85,652	636,434	46,499	93,804	6853	2,277,666	166,409

## Appendix A

**Table A1:** Studies used to calculate relative risks (RRs) for violence against children (VAC)—outcomes.

Study Reference	VAC—Outcomes Relationship
[26]	Sexual Violence—Self-harm Physical Violence—Self-harm
[27]	Physical Violence—Anxiety
[28]	Sexual Violence—Anxiety Physical Violence—Anxiety Emotional Violence—Anxiety Neglect—Anxiety
[29]	Sexual Violence—Interpersonal Violence
[30]	Physical Violence—Interpersonal Violence
[31]	Sexual Violence—Drug Abuse Sexual Violence—Interpersonal Violence
[32]	Sexual Violence—Depression Sexual Violence—Alcohol Abuse Sexual Violence—HIV Physical Violence—HIV Emotional Violence—Alcohol Abuse Emotional Violence—HIV Neglect—Depression Neglect—Alcohol Abuse Neglect—Drug Abuse Neglect—STDs
[33]	Physical Violence—Interpersonal Violence
[34]	Physical Violence—Anxiety
[35]	Sexual Violence—STD
[36]	Sexual Violence—Interpersonal Violence Physical Violence—Interpersonal Violence
[37]	Sexual violence—Depression
[38]	Sexual violence—STD
[39]	Emotional Violence—Self-harm
[40]	Sexual Violence—Interpersonal Violence Physical Violence—Interpersonal Violence
[41]	Sexual Violence—Substance use disorders Physical Violence—Anxiety
[42]	Physical Violence—Alcohol Abuse

**Table A2:** Odd ratios (ORs) for health outcomes associated with childhood violence based on the systematic review.

Type of VAC	SMI	Depression	Anxiety	Alcohol Abuse	Drug Abuse	STDs	HIV	Interpersonal Violence	Self-Harm
Sexual abuse									
Total	-	-	2.8	-	4.9	1.4	-	-	9.3
Males	-	-	-	3.7	-	-	-	2.1	-
Females	-	2.2, 1.5	-	3.9	-	2.2	1.7	3.4, 2.4, 2.3, 2	-
Physical abuse									
Total	-	-	1.9	2, 2.2	-	-	-	-	2.2
Males	-	-	-	-	-	-	-	2.2, 1.4	-
Females	-	-	-	-	-	-	2.1	1.6, 2.2	-
Emotional abuse									
Total	-	-	2.9	-	-	-	-	-	2.4
Males	-	-	-	1.5	-	-	-	-	-
Females	-	-	-	-	-	-	2.0	-	-
Neglect									
Total	-	-	2.5	-	-	-	-	-	-
Males	-	3.4	-	-	2.0	-	-	-	-
Females	-	1.8	-	2.2	-	1.6	-	-	-
Witnessing family violence									
Total	-	-	2.1	-	-	-	-	-	-
Males	-	-	-	-	-	-	-	1.5, 1.7, 2.0, 2.3	-
Females	-	-	-	-	-	-	-	1.6, 1.8, 1.7	-

**Table A3:** Relative risks (RRs) for health outcomes associated with childhood violence based on the secondary analysis of the Cape Area Panel Study.

Type of VAC	SMI	Obesity	Perpetrating Violence	Smoking	Problem Drinking	Illicit Drug Use
Physical abuse	1.409 **	1.348 **	1.184 *	1.103 **	1.124	1.460 **
	(1.065, 1.863)	(1.045, 1.740)	(0.992, 1.414)	(1.009, 1.206)	(0.739, 1.709)	(1.050, 2.031)
Emotional abuse	1.382 **	1.309 **	1.273 **	1.045	1.349 *	1.410 **
	(1.007, 1.896)	(1.045, 1.708)	(1.046, 1.549)	(0.954, 1.146)	(0.891, 2.043)	(1.027, 1.935)

* *p* < 0.10; ** *p* < 0.05.
